# Supplementary material for: PILS-Nir1 is a sensitive phosphatidic acid biosensor that reveals mechanisms of lipid production
Source: J Cell Biol. 2025 Sep 9;224(11):e202405174. doi: 10.1083/jcb.202405174 (PMC12419160; doi:10.1083/jcb.202405174)
Supplement: Table S1 — shows P values from the ordinary one-way ANOVA with multiple comparisons for AUC biosensor data presented in Fig. 1 J. [file jcb_202405174_tables1.docx]

|  | NES-PABD | PASS | NES-flex-PABD | NES-PABDx2 | NESx2-PABDx2 | PILS-Nir1 | Nir2-LNS2 |
| --- | --- | --- | --- | --- | --- | --- | --- |
| PASS  μ = 1.830 | 0.9999 |  |  |  |  |  |  |
| NES-flex-PABD  μ = 1.052 | >0.9999 | 0.9999 |  |  |  |  |  |
| NES-PABDx2  μ = 4.195 | 0.7860 | 0.9435 | 0.7475 |  |  |  |  |
| NESx2-PABDx2  μ = 4.429 | 0.7251 | 0.9114 | 0.6791 | >0.9999 |  |  |  |
| PILS-Nir1  μ = 15.42 | <0.0001 | <0.0001 | <0.0001 | 0.0004 | 0.0005 |  |  |
| Nir2-LNS2  μ = 6.334 | 0.1774 | 0.3493 | 0.1289 | 0.9523 | 0.9739 | 0.0017 |  |
| Nir3-LNS2  μ = 3.329 | 0.9460 | 0.9955 | 0.9350 | 0.9999 | 0.9994 | 0.0002 | 0.7853 |

**Table S1.** P-values from the ordinary one-way ANOVA with multiple comparisons for AUC biosensor data presented in **Figure 1J**. The mean AUC value for each biosensor is shown on the left as “μ". Significant p-values (p < 0.05) are highlighted in gray.
