# Supplementary figures and images for: PILS-Nir1 is a sensitive phosphatidic acid biosensor that reveals mechanisms of lipid production
Source: J Cell Biol. 2025 Sep 9;224(11):e202405174. doi: 10.1083/jcb.202405174 (PMC12419160; doi:10.1083/jcb.202405174)

## SourceDataF2A

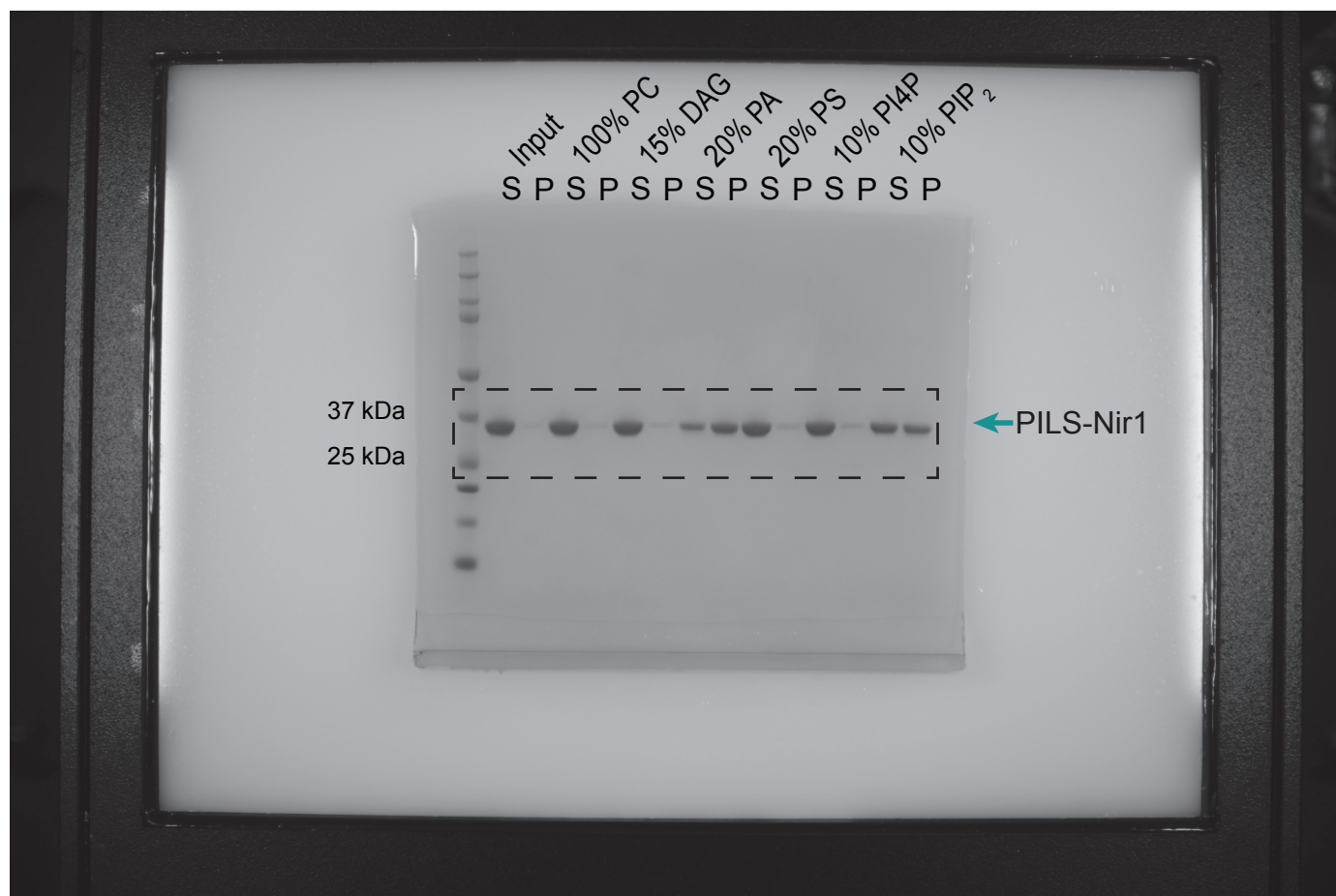

SourceDataF2B

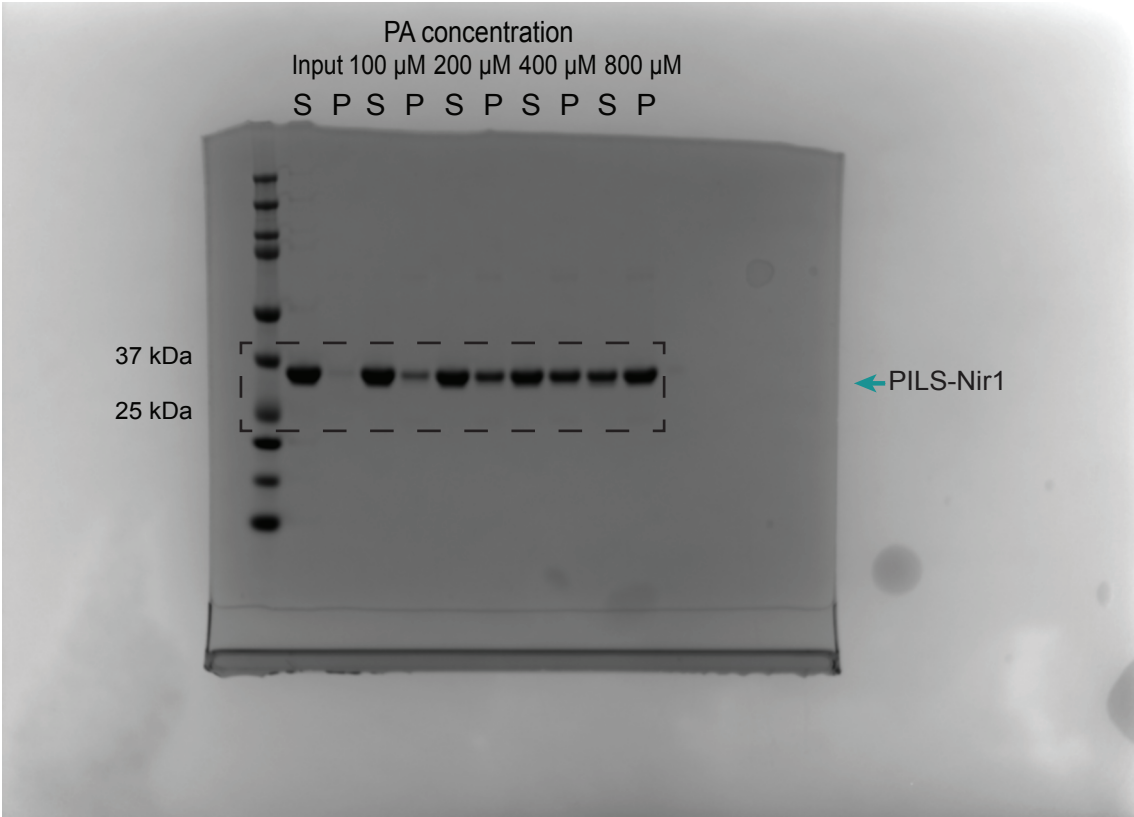

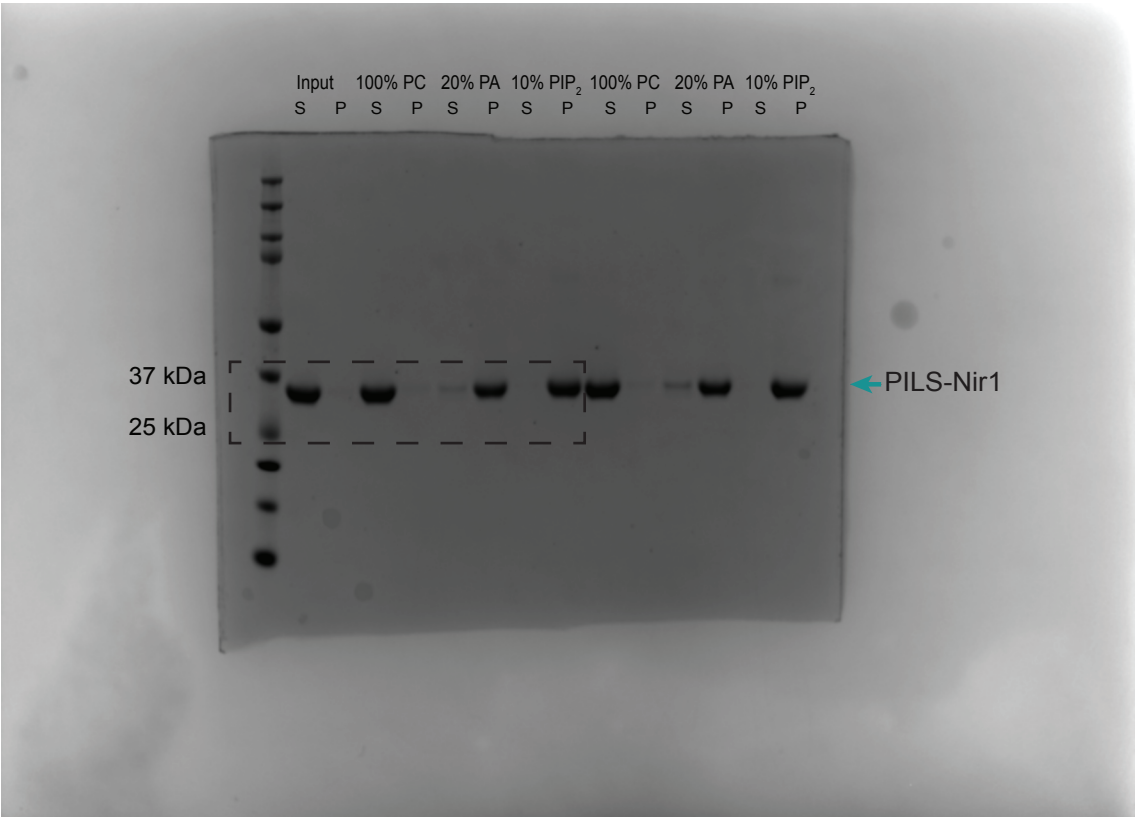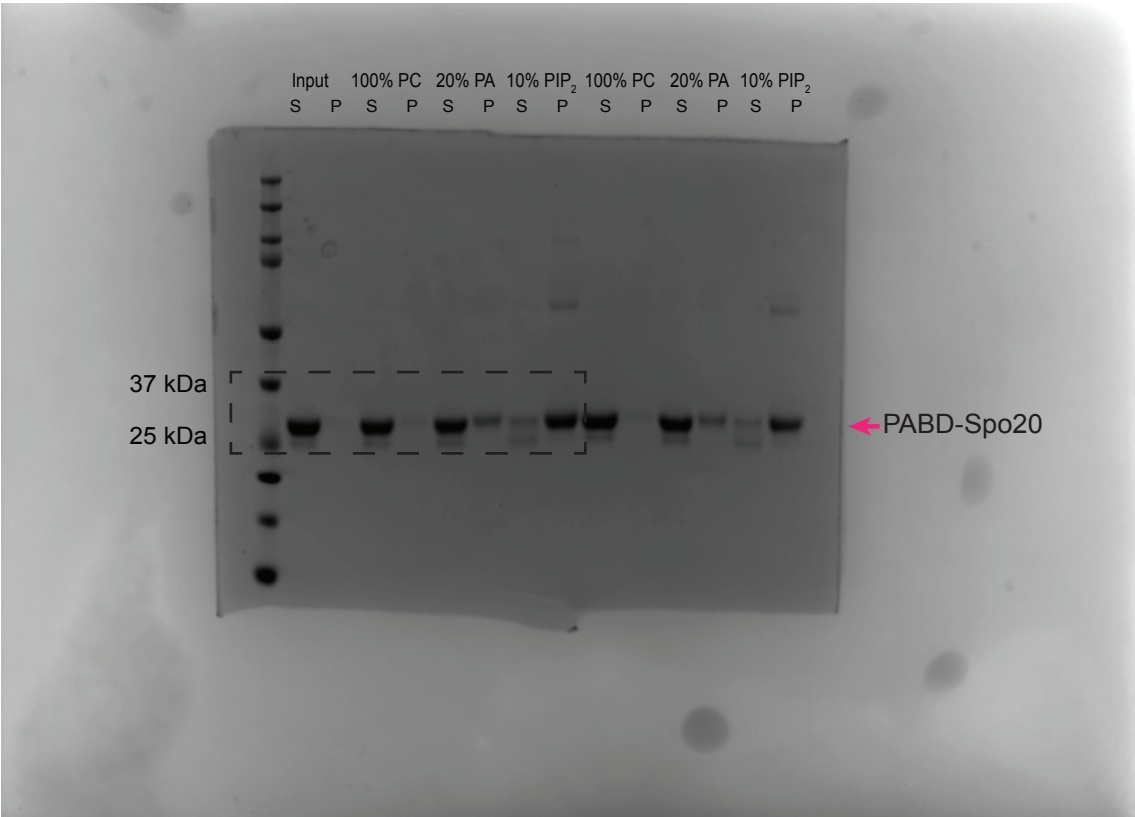

SourceDataF2F

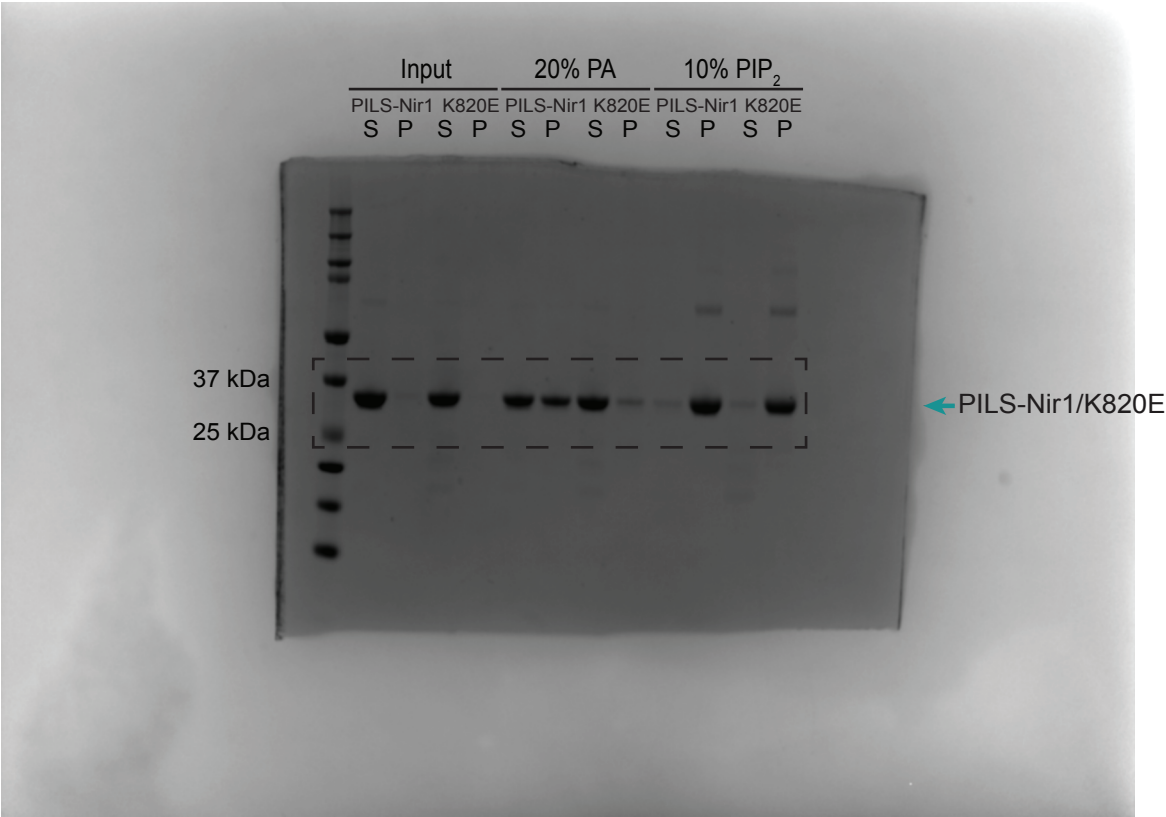

Supplement: SourceData F2 — is the source file for Fig. 2. [file jcb_202405174_sourcedataf2.pdf]

SourceDataFS1B

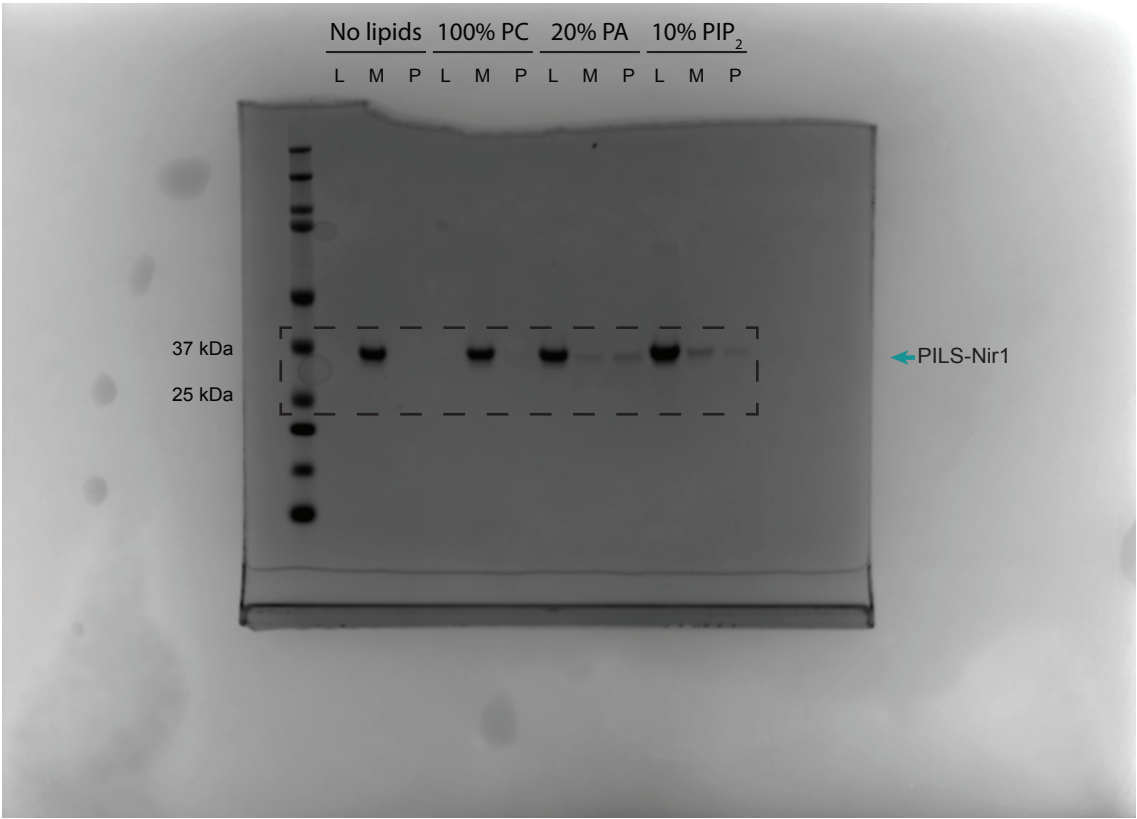

Supplement: SourceData FS2 — is the source file for Fig. S2. [file jcb_202405174_sourcedatafs2.pdf]
